# Identifying and distinguishing of essential tremor and Parkinson’s disease with grouped stability analysis based on searchlight-based MVPA

**DOI:** 10.1186/s12938-022-01050-2

**Published:** 2022-11-28

**Authors:** FuChao Cheng, YuMei Duan, Hong Jiang, Yu Zeng, XiaoDan Chen, Ling Qin, LiQin Zhao, FaSheng Yi, YiQian Tang, Chang Liu

**Affiliations:** 1 College of Computer, Chengdu University, Chengdu, China; 2 Department of Computer and Software, Chengdu Jincheng College, Chengdu, China; 3 Department of Neurosurgery, Rui-Jin Hospital, Shanghai Jiao Tong University School of Medicine, Shanghai, China; 4 Key Laboratory of Pattern Recognition and Intelligent Information Processing, Institutions of Higher Education of Sichuan Province, Chengdu, China

**Keywords:** Grouped stability analysis, Neuroimaging biomarkers, MVPA, Essential tremor, Parkinson’s disease

## Abstract

**Background:**

Since both essential tremor (ET) and Parkinson’s disease (PD) are movement disorders and share similar clinical symptoms, it is very difficult to recognize the differences in the presentation, course, and treatment of ET and PD, which leads to misdiagnosed commonly.

**Purpose:**

Although neuroimaging biomarker of ET and PD has been investigated based on statistical analysis, it is unable to assist the clinical diagnosis of ET and PD and ensure the efficiency of these biomarkers. The aim of the study was to identify the neuroimaging biomarkers of ET and PD based on structural magnetic resonance imaging (MRI). Moreover, the study also distinguished ET from PD via these biomarkers to validate their classification performance.

**Methods:**

This study has developed and implemented a three-level machine learning framework to identify and distinguish ET and PD. First of all, at the model-level assessment, the searchlight-based machine learning method has been used to identify the group differences of patients (ET/PD) with normal controls (NCs). And then, at the feature-level assessment, the stability of group differences has been tested based on structural brain atlas separately using the permutation test to identify the robust neuroimaging biomarkers. Furthermore, the identified biomarkers of ET and PD have been applied to classify ET from PD based on machine learning techniques. Finally, the identified biomarkers have been compared with the previous findings of the biology-level assessment.

**Results:**

According to the biomarkers identified by machine learning, this study has found widespread alterations of gray matter (GM) for ET and large overlap between ET and PD and achieved superior classification performance (PCA + SVM, accuracy = 100%).

**Conclusions:**

This study has demonstrated the significance of a machine learning framework to identify and distinguish ET and PD. Future studies using a large data set are needed to confirm the potential clinical application of machine learning techniques to discern between PD and ET.

## Introduction

Essential tremor (ET) and Parkinson’s disease (PD) are two of the most common movement disorders, with ET being the most common tremor disorder and PD being one of the most common neurodegenerative diseases like Alzheimer’s disease. Although tremor is the primary symptom for both ET and PD, there are many differences in the signs and symptoms of the two disorders. ET often happens bilaterally and is primarily seen during action while PD’s tremor commonly occurs at rest and presents unilaterally and later progresses to both sides of the body. Furthermore, the primary symptom of ET is tremor while the cardinal symptoms of PD not only contain tremor, but also include bradykinesia, rigidity and gait/balance problems. Meanwhile, a longstanding clinical literature has demonstrated an association between ET and PD, which have shown ET might develop PD. But whether ET is a risk factor for PD still remains a controversial issue [1, 2]. Nonetheless, these differences are not easily recognized and misdiagnoses often occurred due to the same symptoms in clinical diagnosis, especially during their initial stages. It is reported that the misdiagnosis rate for ET and PD can be higher than 25% [3].

In order to differentiate ET and PD, previous studies mainly focused on tremor amplitude and spectral analysis of surface electromyography (EMG) based on pattern classification technologies [4–6]. The balance and gait characteristics, collected by body-worn inertial motion unit sensors, also have been with machine learning techniques [7]. But the classification results of these methods highly depend on the robustness of collected signals. Furthermore, whether these diseases have an impact on brain alterations still keeps unknown.

Recently, neuro-imaging techniques have provided insights on the brain structural/functional abnormality of various diseases. Some studies have paid attention to revealing the differences between PD patients and normal controls (NCs) [8, 9] or ET patients and NCs [10]. Only few studies have looked into the differences and associations of their two diseases from the view of structural imaging [10–12]. Benefiting the statistical parametric mapping and voxel-based morphometry (VBM) technique, the differences between PD and ET has been investigated through the comparison of two kinds of VBM processing [10]. Compared with healthy control, ET patients had shown cerebral and cerebellar atrophy in scattered areas, while the decrease of the volumes in the lentiform nucleus, the insula, the middle frontal gyrus, and the cerebellar vermis had been revealed in PD patients. Horizontal VBM comparisons also have indicated that ET and PD caused specific patterns of brain volume alterations including the thalamus, the middle temporal gyrus, the middle frontal gyrus, the cerebellum posterior lobe and the insula. The white matter (WM) of PD and ET has been explored, and it has been pointed out that white matter microstructure differences in ET are mainly located in cerebellar peduncles and thalamo-cortical visual pathways [11]. It has been reported that the brain changes of gray matter are mainly related to basal ganglia for PD and NC, right superior temporal gyrus, right angular gyrus, right inferior temporal gyrus, and left middle temporal gyrus for ET and NC [12]. It also has been found that significant group differences and asymmetric morphologic changes in the inferior opercular gyrus of white matter between ET and PD. However, these existing research work is limited which primarily have contributed to analyze the group differences of ET and PD in term of conventional mass-univariate analysis and is failing to reveal disease effects. Until now, there is still a lack of stable neuroimaging biomarkers and is not able to identify and distinguish PD and ET at the individual level.

To date, machine learning technique, also called multi-voxel pattern analysis (MVPA) in the field of neurobiology, has been advocated to identify neuro-imaging markers to classify different groups at the level of individual subjects and provide more complementary information on the pathophysiological framework of the human brain. Instead of statistical inference based on single voxel for mass-univariate analyses, multi-voxel pattern analysis (MVPA) considers the dependencies of different voxels and consistently excavates the most discriminative information from different data groups, which is helpful to reduce the disturbance of noise voxels and increase the classification power of machine learning model. In the last few years, a growing number of studies have been processed with the use of MVPA to analyze neuroimaging data on various diseases, including schizophrenia, autism spectrum disorder and attention-deficit/hyperactivity disorder [13, 14]. We have distinguished and identified neuro-imaging markers of PD from NC with MVPA [15]. To the best of our knowledge, we have investigated firstly PD and ET based on machine learning and achieved satisfactory classification performance [16]. To avoid the curse of dimensionality and enhance computational efficiency, machine learning models often reduce the dimensionality of high-dimensional neuroimaging data as the data preprocessing step [17, 18], and then classify the reduced data, i.e., features with the classifiers, such as support vector machines (SVM), which has been proved widely to have the excellent generalization capability for new samples [19, 20].

As a new branch of a broader family of machine learning methods, deep learning has gained more popularity during the most recent years. Deep learning models can learn complex features from hierarchical network structure rather than handcrafted features in traditional machine learning. The successful applications of deep learning are numerous in neuroscience to reveal imaging signatures for various diseases and disorders and identify individual brains and brain states. It is notable to point out that deep learning requires a very large amount of samples available for training due to the risk of overfitting and poor generalization. These investigations have been made to advance the diagnosis of various diseases with the help of neuroimaging.

Differentiating from other machine learning studies of computer vision, such as natural image, remote sensing image, neuroscience has a long-standing interest to localize structural and/or functional abnormality induced by diseases. Therefore, neuroimaging-based machine learning models not only pursue to discriminate patients from normal controls with the accurate classification results, but also are capable of identifying the contribution of brain regions and comprehend the relationship between the models and neural or disease processes. Consequently, a standard protocol of machine learning is required to enhance the interpretability of learning models and improve the reproducibility of machine learning model. Therefore, a comprehensive framework has been developed to unify the analysis procedure of machine learning in the field of neuroimaging, including model-, feature- and biology-level assessment [21]. The model-level assessment evaluates the discriminative ability of a learning model in term of the different groups of neuroimaging data. The feature-level assessment aims to identify the importance of features based on a learned model and the derived results are helpful to understand the underlying pathophysiological of disease. The biology-level assessment suggests the neuroscientific plausibility of the findings based on machine learning according to the horizontal comparison with previous studies. Actually, most MVPA research work can be regarded as the model-level assessments of this framework. Since existing approaches mainly accounts for the discriminant capability of voxels to treat these adjacent voxels (clusters) as biomarkers but neglects local correlations based on the structural/functional brain atlases. For example, some studies have defined adjacent voxels using spherical volumes with a small radius (e.g., 2 mm) or *N*-connected neighborhoods (26-connected) even if these voxels belong to different structural/functional brain regions. As a result, simply emphasizing the discrimination of spatial location may harm the interpretation of the results. In tackling this limitation [22, 23], have divided voxels into groups in term of prior structural segmented brain atlas to alleviate this limitation.

In this paper, we have proposed a MVPA framework and attempted to identify neuro-imaging markers of ET and PD and distinguished ET from PD based on structural MRI. The proposed MVPA framework followed the unified protocol in [21] and intended to implement the identification and classification of different group from model-, feature- and biology-level assessment, respectively. In the model-level assessment, we have completed searchlight-based MVPA to identify the brain alterations of different groups roughly. And then, we have incorporated prior structural brain atlas to divide these brain changes into different groups and processed a group-wise stability analysis based on the permutation test to reveal and refine the brain biomarkers from the aspect of the feature-level assessment. Finally, we have made a discussion about these results trying to comprehend the working mechanism of the model with the support of previous neurobiology findings from the level of biology. Our findings have provided further insight into structural alteration and automatic diagnosis of ET and PD.

## Materials

### Subjects

In this study, we have enrolled three number-balanced groups, including an ET group of 15 subjects, a PD group of 16 subjects and a NC group of 17 subjects. All study procedures and ethical aspects were subject to approval by the institutional reviewer board. Written informed consent was obtained from all subjects. All patients and the healthy control subjects were examined by neurologists with more than ten years of experience in movement disorders. The healthy control subjects had no history of neurologic or psychiatric diseases, with normal neurological examinations. The demographics information about subjects and average total intracranial volume (TIV) are summarized in Table 1.

**Table 1:** Demographics information and TIV

	NC	ET	PD
Gender (M/F)	9/8	6/8	12/4
Age	$53.294\pm 10.575$	$56.64\pm 12.029$	$69.13\pm 8.899$
TIV	$1360.086\pm 135.087$	$1297.322\pm 160.517$	$1426.902\pm 180.805$

Images were acquired on a 3T MRI scanner (GE DISCOVERY MR750) at the MRI Research Center of University of Electronic Science and Technology of China. High-resolution MRI data were acquired using a 3D T1-weighted spoiled gradient echo sequence (T1-3D FSPGR) with the following parameters: TR = 6.008 ms, flip angle = 9°, FOV = 25.6 cm $\times$ 25.6 cm, matrix = 256 $\times$ 256, slice thickness = 1 mm, no gap, 152 slices. During scanning, foam padding and ear plugs were used to reduce head motion and scanning noise, respectively. According to visual inspection, T1 images with obvious motion artifacts have been removed from dataset.

### Magnetic resonance imaging

**Fig. 1:**
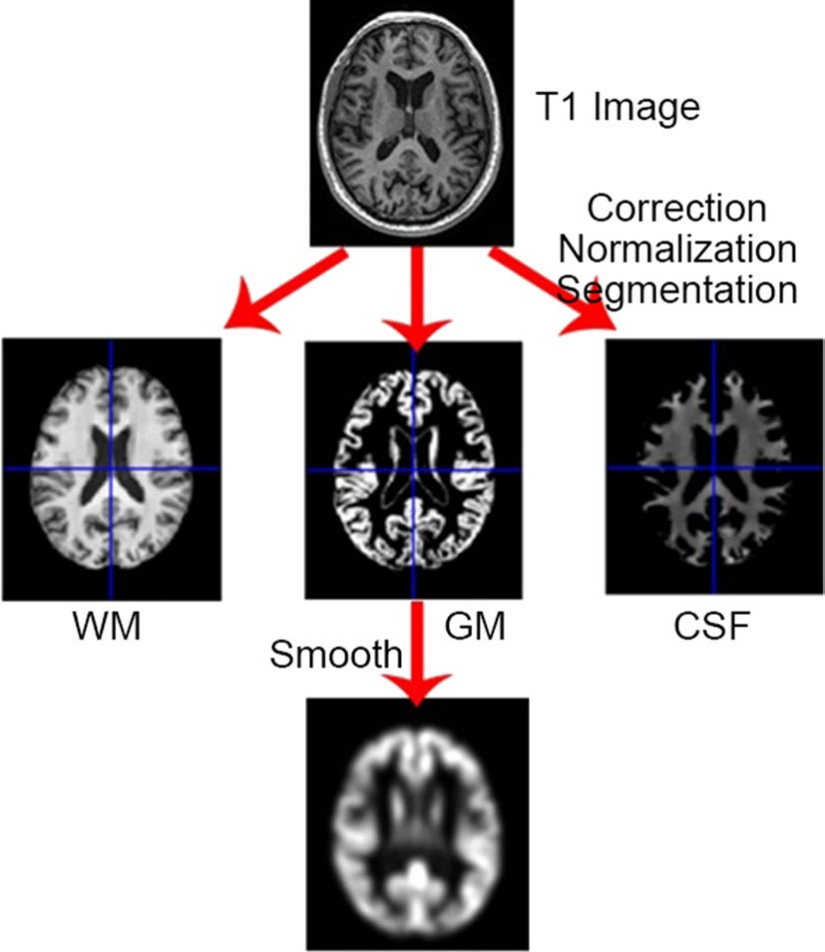
The preprocessing of MRI images

Preprocessing of MRI images was done using the SPM8 package (Welcome Department of Cognitive Neurology, London, UK (http://www.fil.ion.ucl.ac.uk/spm) and the VBM8 (voxel-based morphometry) toolbox (http://dbm.neuro.uni-jena.de) running under Matlab R2014a (Mathworks) . All T1-weighted images were corrected for bias-field inhomogeneities, then spatially normalized and segmented into gray matter (GM), white matter (WM) and cerebro-spinal fluid (CSF) within the same generative model. The segmented procedure has further applied adaptive maximum a posterior and estimations [24] and hidden Markov random field model [25] by accounting for partial volume effects [26]. In this study, GM images were included to investigate abnormal brain regions. Subsequently, spatial smoothing (Gaussian kernel with 6 mm full-width at half-maximum) was performed on GM and WM images to remove noise [27]. The preprocessing procedure is shown in Fig. 1.

## Results

### Statistical analysis

**Fig. 2:**
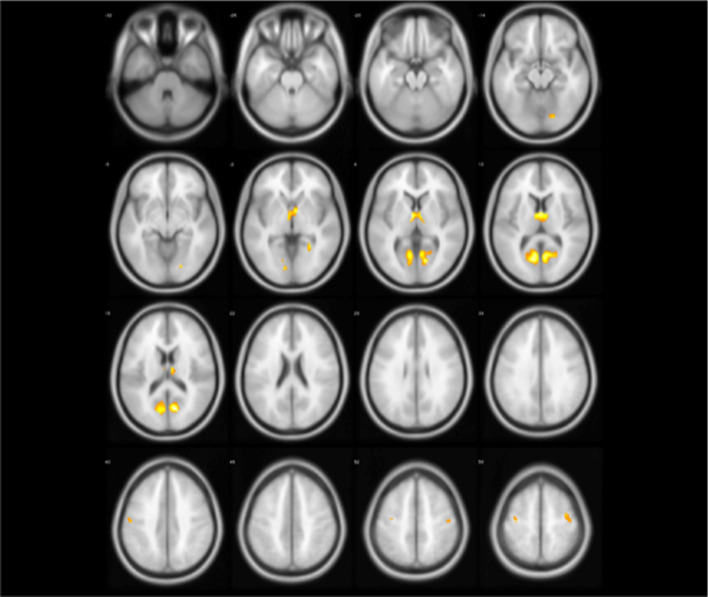
The group differences (yellow regions) identified by two-sample test (NC > ET)

**Fig. 3:**
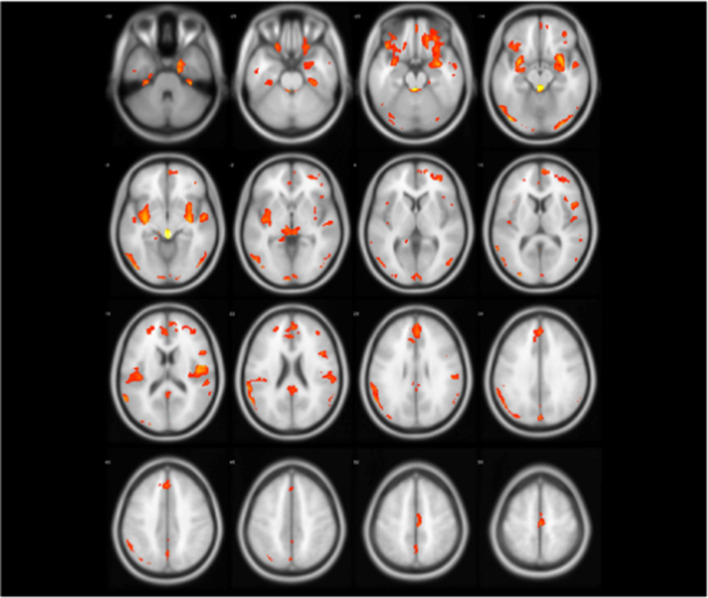
The group differences (orange regions) identified by two-sample test (NC < ET)

**Table 2:** The abnormal brain regions of ET identified by statistical analysis

Cluster ID	Brain regions	BA	Peak MNI coordinates			Cluster size (voxels)	*T*
			*x*	*y*	*z*		
	*NC > ET*						
1	LING.R	18	19.5	− 79.5	− 9	90	5.6986
2	CAL.L,LING.L	18,30,31	− 12	− 70.5	12	1375	7.5639
3	THA.R,THA.L	–	6	− 7.5	3	994	7.2812
4	CAL.R,LING.R	18,30,31	10.5	− 69	16.5	1185	7.4777
5	PoCG.L	4	− 54	− 16.5	39	62	4.4564
6	PRreCG.R,SFGdor.R	6,4	37.5	− 7.5	58.5	146	4.4379
	*NC < ET*						
1	Cerebelum9.R	–	6	− 46.5	− 42	94	4.0545
2	ORBinf.R,ORBmid.R, ORBsup.R,AMYG.R, PHG.RINS.RHIP. RREC.R,TPOsup.R,OLF.R	13,22,28,47	31.5	6	− 12	3763	8.3214
3	FFG.R,PHG.R	35	34.5	− 28.5	− 30	331	6.7992
4	ITG.L	–	− 49.5	− 9	− 25.5	72	3.9479
5	Vermis3,LING.L, Vermis12,Cerebelum3.L	–	6	− 31.5	− 7.5	1005	13.619
6	ORBinf.L,PUT.L,TPOsup.L, AMYG.L,HIP.L,ORBsup.L,STG.L	47	− 30	3	− 13.5	2667	8.4816
7	STG.R,MTG.R	21	54	− 4.5	− 9	690	6.5337
8	SFGmed.L,SFGmed.R, ACG.L,ACG.R,REC.R	9,10,32,6,11	1.5	42	31.5	2262	6.0785
9	IOG.L,CAL.L,MTG.L,ITG.L, MOG.L,FFG.L,Crus1.L,LING.L	37,18,19,17	− 42	− 18	− 15	1567	8.958
10	IOG.R,ITG.R,MTG.R, LING.R,MOG.R	18,19,37	31.5	− 88.5	− 13.5	742	6.2051
11	STG.L	–	− 49.5	− 10.5	− 6	56	4.4417
12	MFG.R,SFGdor.R	10	34.5	46.5	6	722	6.1841
13	MOG.L	–	− 28.5	− 91.5	9	162	7.628
14	SMG.L,ROL.L,ANG.L, IPL.L,MTG.L,MOG.L	39,40,13,19,22	− 60	− 57	21	2240	6.4548
15	IFGoperc.R	44	45	12	22.5	503	6.1158
16	ROL.R,SMG.R,STG.R	13.43	46.5	− 15	16.5	1296	7.3077
17	MFG.L	–	− 27	40.5	16.5	236	4.6633
18	PCG.L,PCG.R	23	0	− 51	15	476	4.8023
19	ANG.R,STG.R	22	63	− 46.5	18	121	4.0803
20	PCUN.L	–	0	66	37.5	114	4.568
21	PCUN.L,PCUN.R	–	0	− 54	51	129	4.4936
22	SMA.R,PCL.L	6	3	− 18	64.5	905	6.2211

According to statistical analysis, it is worth noting that there is no gray matter difference between PD patients and normal people. However, we can find that gray matter volume of ET has changed widely compared with NC as shown in Table 2, Figs. 2 and 3, which involved the frontal lobe, parietal lobe, temporal lobe, occipital lobe, insula and limbic system. For some brain regions, GM has decreased including bilateral partial lingual gyrus, bilateral calcarine cortex, bilateral thalamus, right precentral gyrus, left postcentral gyrus and right dorsolateral superior frontal gyrus; meanwhile, GM has increased in most brain areas. Interestingly, in the bilateral lingual gyrus and right dorsolateral superior frontal gyrus, partial gray matter atrophy and partial gray matter increase. It is not difficult to find these abnormal brain regions involve motor Brodmann’s area 4 (BA4), BA6, visual (BA17, BA18, BA19, BA37), emotion and cognition (BA32), auditory processing and language perception (BA22), memory (BA28, BA35), semantic processing of language spatial information (BA39, BA40, BA44).

### Machine learning analysis

#### The abnormal brain regions of ET

**Fig. 4:**
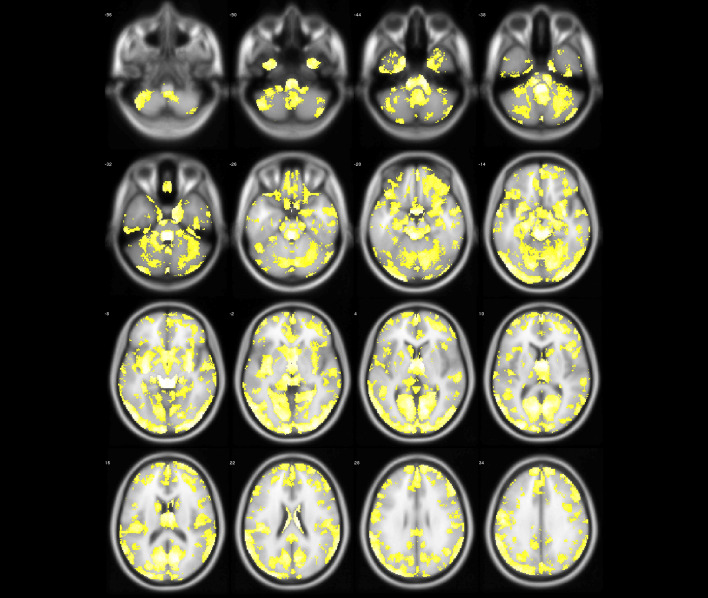
The abnormal brain regions (yellow regions) of ET identified by model-level assessment (accuracy >70%, cluster size > 50)

**Fig. 5:**
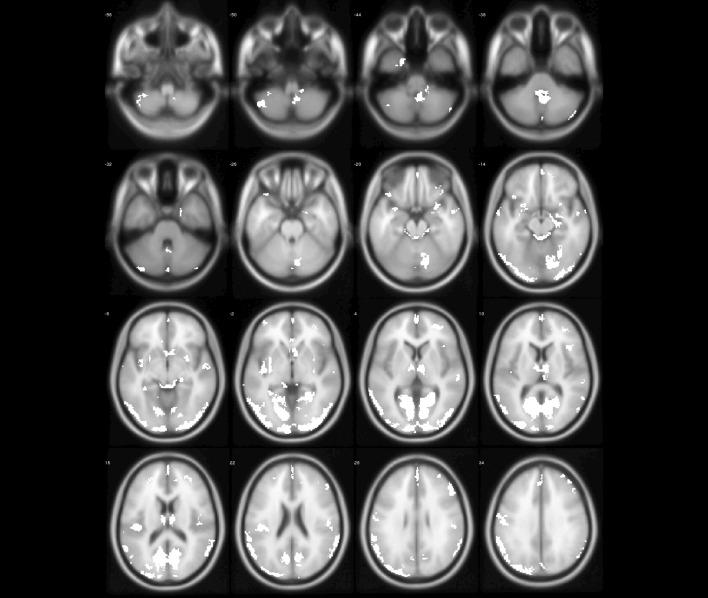
The abnormal brain regions (white regions) of ET identified by feature-level assessment (accuracy > 70%, cluster size > 50, permutation test $p<0.01$)

**Table 3:** The abnormal brain regions of ET identified by feature-level assessment

Cluster ID	Brain regions	BA	Peak MNI coordinates			Cluster size (voxels)	ACC (%)
			*x*	*y*	*z*		
1	Cerebelum3.L,Vermis3,LING.R	–	0	− 37.5	− 21	427	100
2	IOG.R,CAL.R,MOG.R,LING.R	18,19	30	− 93	− 9	924	98.33
3	MOG.L,MTG.L	37,19	− 52.5	− 67.5	− 1.5	326	100
4	CAL.L,MOG.L,SOG.L	17,18	− 6	− 99	− 7.5	373	100
5	PUT.R	–	33	− 13.5	− 6	81	96.67
6	PUT.L	–	− 31.5	− 10.5	− 6	124	100
10	CAL.L,LING.L	30,31,18	− 10.5	− 69	10.5	1167	100
11	CAL.R,LING.R	31,30,18,23	15	− 69	1.5	1150	100
12	THA.R,THA.L	–	9	− 7.5	1.5	556	100
14	MOG.L	19	− 25.5	− 93	7.5	336	100
15	ANG.L,IPL.L,MOG.L	19,7	− 40.5	− 75	42	610	100
16	PoCG.L	–	− 54	− 16.5	36	125	100

Figures 4 and 5 show abnormal brain regions of ET obtained by model-level and feature-level assessment, respectively. It is not difficult to find that the number of abnormal brain regions has decreased after feature-level assessment to avoid the influence of structural boundaries of brain regions. Meanwhile, the abnormal brain regions after feature-level assessment have covered most of brain regions found by statistical analysis. In addition, feature-level assessment also has found the abnormality of bilateral cuneus, bilateral superior occipital gyrus and right putamen. It is worth noting that even if both machine learning technique and statistical analysis have found the differences in the cerebellum, machine learning with feature-level assessment also found more extensive differences in the cerebellum. In particular, Table 3 also has listed the clusters whose classification accuracy of ET and NC is greater than 90%, indicating that these brain regions corresponding to these clusters are significantly discriminative.

#### The abnormal brain regions of PD

**Fig. 6:**
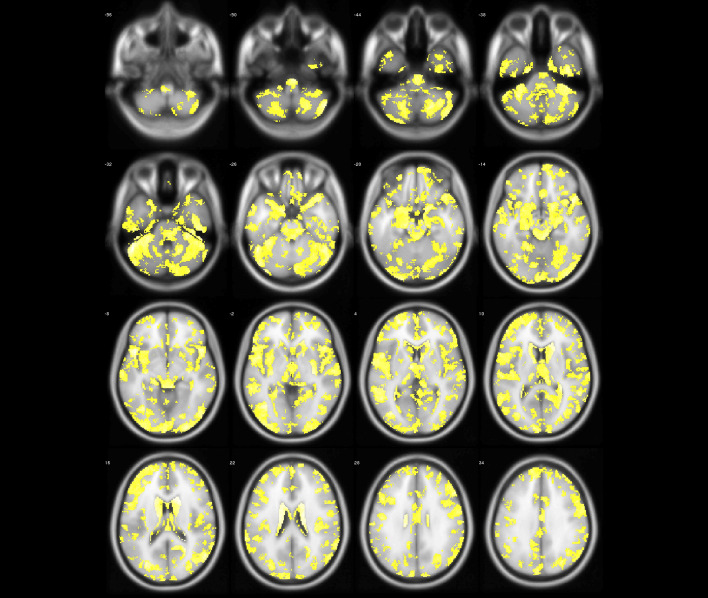
The abnormal brain regions (yellow regions) of PD identified by model-level assessment (accuracy > 70%, cluster size > 50)

**Fig. 7:**
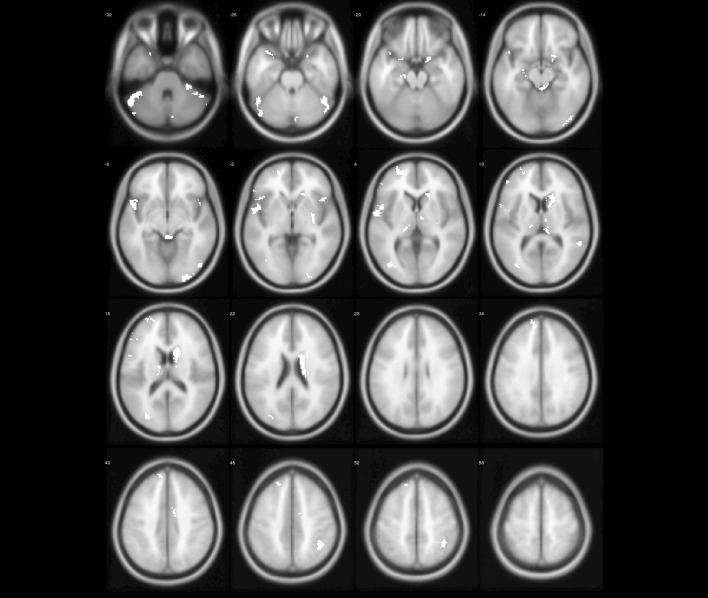
The abnormal brain regions (white regions) of PD identified by feature-level assessment (accuracy > 70%, cluster size > 50, permutation test $p<0.01$)

**Table 4:** The abnormal brain regions of PD identified by feature-level assessment

Cluster ID	Brain regions	BA	Peak MNI coordinates			Cluster size (voxels)	ACC (%)
			*x*	*y*	*z*		
1	CRUS1.L	–	− 49.5	− 52.5	− 30	100	98
2	CAU.R	–	19.5	15	15	388	100

Figures 6 and 7 show PD abnormal brain regions obtained by model-level assessment and feature-level assessment, respectively. The abnormal brain regions detected by feature-level assessment are different from statistical analysis,involving in cerebellum, temporal lobe, frontal lobe, occipital lobe, parietal lobe, and limbic system. Table 4 lists detected clusters with classification accuracy greater than 90% for PD and NC. It can be seen that CrusI in cerebellum and right caudate nucleus have achieved superior classification performance. Furthermore, as shown in Figs. 5 and 7, we can find that the abnormal brain areas of ET and PD partially overlap after the same feature-level assessment, but the gray matter of ET is more widespread than PD.

#### The distinguishing of ET from PD

In order to verify the robustness and classification performance of abnormal brain regions of ET and PD identified in the feature-level assessment stage, the paper has applied five machine learning models including RandomForest, Stochastic Gradient Descent (SGD), Bagging, Gaussian Naive Bayes (GsNB) and Principal Component Analysis followed Support Vector Machine (PCA + SVM) to classify ET from PD based on these abnormal brain regions. Moreover, the paper also has compared the classification performance on smoothed gray matter with these four models directly. The classification procedure has conducted based on fivefold cross-validation with 10 iterations, on the other hand, the paper has employed Optuna to optimize hyper parameters of machine learning models.

**Fig. 8:**
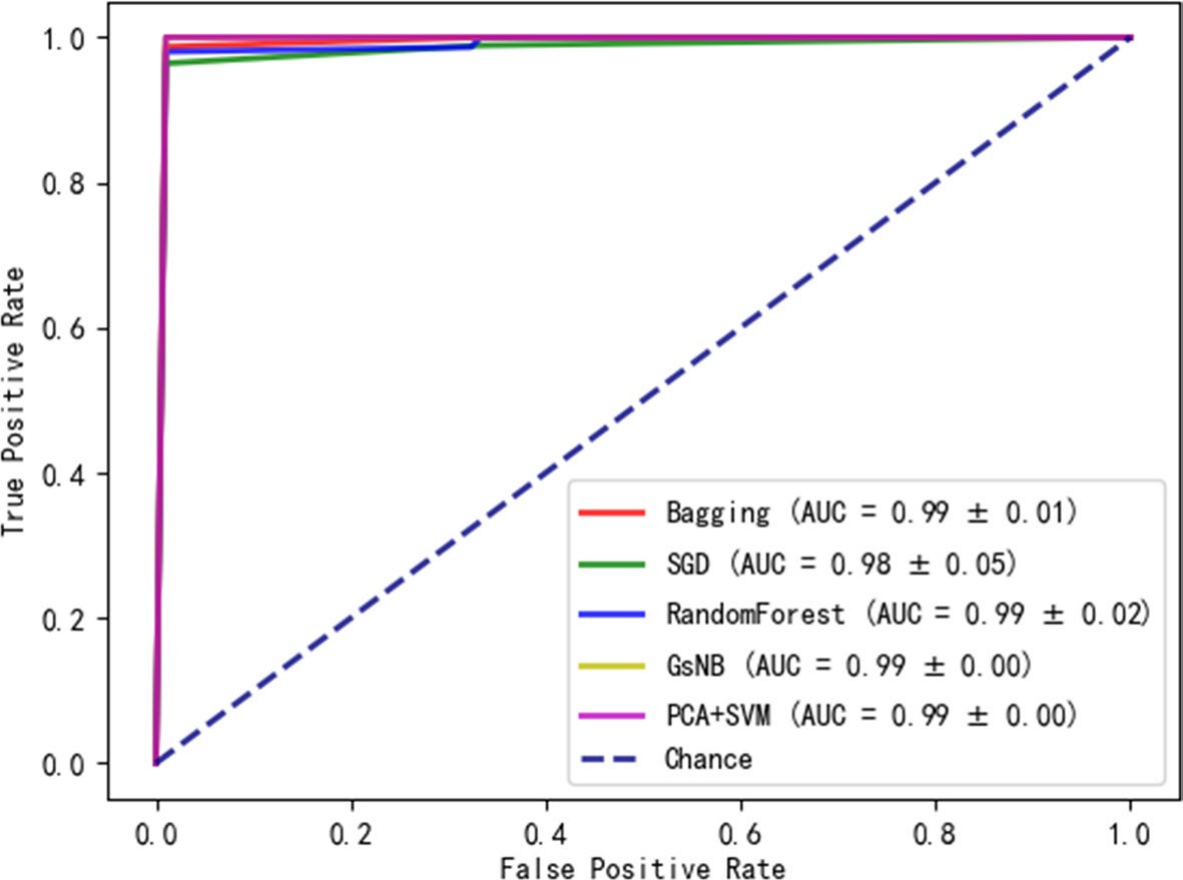
The ROC curve of different methods on GM

**Table 5:** The classification performance based on machine learning

Method	The classification directly			The classification with our framework		
	ACC (%)	SEN (%)	SPE (%)	ACC (%)	SEN (%)	SPE (%)
RandomForest	95.3	95.7	95	96	96.2	96
SGD	94.3	93.5	95.3	95.7	94.3	97.7
Bagging	92.7	93.7	91.3	95.7	98.8	92.3
GsNB	90	93.3	85.7	99.9	100	98.7
PCA+SVM	95.7	95.8	96	100	100	100

The classification results are shown in Table 5. We can achieve superior performance to distinguish ET from PD based on both smoothed gray matter and abnormal brain regions and PCA + SVM has obtained the best classification results. But, the classification of abnormal brain regions can not only improve the performance efficiently, but also reduce the computational cost. The paper has also provided receiver operating characteristic (ROC) curves of different machine learning methods, as shown in Fig. 8. It can be seen that different machine learning models can achieve satisfactory classification performance. Due to significant classification performance of these abnormal brain regions, they can be considered as neuroimaging biomarkers of ET and PD.

## Discussion

The paper has applied statistical analysis and machine learning methods to identify the biomarkers of ET and PD. Based on statistical analysis, widespread alterations have been found in ET patients and there are no gray matter differences in PD patients. While the alterations have been found for both ET and PD patients based on machine learning and the abnormal brain regions are overlapped, which is consistent with the findings in [10], and is helpful to explain the difficulties to discriminate ET and PD. However, it is notable that the abnormal brain regions of ET are broader than PD, which is consistent with [28]. From the perspective of functional imaging, it has been pointed out that ET can lead to subtle morphological changes of multiple neural networks, involving the cerebellum, brainstem, frontal lobe and thalamus [28].

Clinically, it is considered that PD and ET are two different diseases. But it is easy to misdiagnose PD as ET due to their same clinical symptoms, and some patients with ET will be transformed into PD [29]. Autopsy and pathological studies have shown that the loss of melatonin dopamine neurons and increased iron deposition in the basal ganglia have happened in PD, so PD mostly involved the abnormalities of the basal ganglia. However, due to the limited sample size, the brain markers of ET cannot be identified at present. However, radiotracer imagings have shown that patients with ET retain dopaminergic energy to a large extent, and most studies also have shown that the measurement of iron content in substantia nigra and neuromelanin in patients with ET is not significant. Therefore, dopaminergic and iron imaging has shown that there is no substantial overlap between the pathophysiology of ET and PD.

Due to non-invasive advantages of neuroimaging, researchers have carried out various studies on ET and PD based on neuroimaging to clarify the neuroimaging markers of the two diseases and explore their pathological mechanism. However, the findings based on structural images are inconsistent, and even contradicted [30], it has been pointed out that the findings of gray matter abnormalities in ET are unreliable [31]. On the one hand, T1 structural image has high image contrast in cortex and basal ganglia, while image contrast decreases due to the increase of iron content in the midbrain area for PD patients, so it is impossible to accurately detect brain abnormalities; On the other hand, it has been believed that ET is not only a simple disease, but a general term of a class of diseases with individual heterogeneity. Therefore, heterogeneity in data often leads to inconsistent findings.

Although the pathogenesis and neural mechanism of PD and ET are still not clear, a large number of studies have found that some brain regions might be related to their common symptom—tremor, and the most consistent finding is the cerebellum. Compared with the inconsistent results in traditional MRI studies, most findings based on diffusion tensor imaging (DTI) have pointed out the microstructure changes of cerebellar. As a consequence, cerebellar abnormalities have been considered as the direct cause of tremor. The cerebellar abnormalities in patients with ET and PD were both found in [9, 30], but it is still controversial whether the gray matter volume of the cerebellum increases or decreases. Although statistical analysis in the paper did not find cerebellar abnormalities in PD patients, it was found that the cerebellar gray matter of ET patients increased, which is consistent with [32], which has believed that the increase of cerebellar for gray matter in ET patients may be the compensatory result of cerebellar dysfunction. In addition, machine learning analysis in the paper found that both ET and PD patients had cerebellar abnormalities, and the larger area of cerebellum abnormalities in ET patients also partially supported the point that cerebellar abnormalities existed in ET and PD.

In order to further explore the role of the cerebellum, it has been found that missing Purkinje cells in the cerebellum for most ET patients through autopsy [33]. The possible reason is that Purkinje cells are inhibitory neurons and participate in the processes of motor control and learning. Therefore, the loss of Purkinje cells may lead to excessive activity of the cerebellum, which results in tremor. It has also been suggested that the pathological changes of locus coeruleus lead to the changes of Purkinje cells, because Lewy bodies are concentrated in locus coeruleus, and locus coeruleus are the important source of noradrenaline in the brain and has a main efferent connection with Purkinje cells in the cerebellum [33]. On the other hand, it has been pointed out that cerebellar abnormalities may be related to cognitive impairment from the perspective of neuropsychological symptoms, and this kind of abnormality may be caused by the interruption of communication pathway from cerebellum to prefrontal cortex through parietal lobe, temporal lobe and limbic system [34]. Especially through machine learning method, it was found abnormalities in CrusI for both ET and PD. It has been pointed out that most areas of CrusI are related to default mode network and control network [9], while the default mode network is responsible for emotion, self reference and projection, so patients with PD and ET will appear depression and cognitive impairment. It has also been pointed out that the cognitive decline of ET patients was faster than that of age-matched healthy subjects [35].

Based on machine learning, the paper also found that an abnormality of lobule VIII for ET, but PD did not, which is consistent with [36]. Through correlation analysis between the clinical tremor score and the gray matter volume of each region of the cerebellum, it has been found that tremor severity of ET is negatively correlated with the volume of lobule VIII [36]. Moreover, compared with PD, more extensive cerebellar regions have been found for ET, including lobules 3, 4, 5, 7, 8, 9, crusI and vermis 3. Among them, lobules 4, 5, 6 and 8 involved sensorimotor regions, which not only showed that tremor symptoms of PD and ET were related to the sensorimotor function of cerebellum, but also explained that the tremor symptoms of ET were related to a wider range of sensory dysfunction.

In the past 10 years, most studies have found abnormalities in different parts of the cerebellum in patients with ET [37, 38]. At present, it is necessary to clarify whether ET affects the whole cerebellum or only some specific areas of the cerebellum. Because the anterior part of the cerebellum is related to movement, its posterior part is related to cognition [33] . In particular, neural activation patterns between frontal lobe, cerebellum and dentate nucleus are related to executive function and language working memory [39]. It has been pointed out that ET may first affect the anterior part of the cerebellum, and then affect the posterior part of the cerebellum with the development of the disease, resulting in the defect of cognitive function [33]. However, it is unclear whether ET patients with severe cognitive impairment are accompanied by more severe abnormalities in the posterior cerebellum. However, it has been suggested that cerebellar cortical abnormalities may be potential neuroimaging markers of ET [37].

In order to explain the occurrence and duration of tremor, it has been believed that the tremor signal of ET patients originated from the cerebellum, transmitted to the relay station-thalamus, and finally reached the motor cortex of the forebrain [40]. This pathway is also known as the cerebello-thalamo-cortical pathway (CTC). Ref. [41] also has supported that the abnormality of CTC is involved in the generation and transmission of tremor. For PD, it has been suggested that the tremor in PD patients is related not only to the pathophysiological changes of the CTC motor circuit, but also to the basal ganglia [42]. While they believed that the tremor of PD patients first occurred in the basal ganglia and then spread to the CTC circuit through the motor cortex [43]. The paper based on statistical analysis found that the atrophy of bilateral thalamus and increased gray matter volume of cerebellar in ET. Meanwhile, machine learning in the paper also found that the abnormalities of bilateral thalamus in both ET and PD, which may confirm that the compensatory effect on the CTC circuit and the occurrence of tremor is related to the abnormality of the CTC circuit. Moreover, this paper also found abnormalities of basal ganglia of ET and PD (such as putamen and caudate) with machine learning, which is consistent with [12, 44]. In addition, the increase of gray matter volume of MTG in the paper is partially consistent with [10], which has proposed that the function of MTG involved motion perception and eye movement, and some ET patients have long-term head tremor, which may need to be supplemented by maintaining visual stability. As a consequence, the MTG of ET may be continuously activated, which lead to increasing gray matter volume to keep coordination and balance between head and eyes.

Recently, the tremor behavior of ET patients with gamma aminobutyric acid (GABA) hypothesis has been tried to explain [45]. GABA is a molecule produced by the brain. It mainly acts as a chemical messenger or neurotransmitter to control some activities of the brain. GABA can regulate the excessive excitation of brain nerve cells, so it is also called inhibitory neurotransmitter. In the CTC, nerve cells in the cerebellum can produce and release GABA, which are responsible for regulating and controlling movement. For ET patients, the abnormal cerebellar leads to the reduction of GABA molecules, which makes the activity of deep cerebellar neurons abnormally excited, and leads to the acceleration of the rhythm of the thalamus and its pathway and produces tremor. However, the abnormal evidence of the GABAergic neurotransmitter system in patients with ET obtained with positron emission tomography (PET) and magnetic resonance spectroscopy (MRS) is limited.

In addition, the paper has found increased gray matter related to visual processing function, such as BA17, BA18, BA19, BA21 and BA37. It has long been suspected that the increase of visual information will lead to the severity of tremor, this hypothesis has been confirmed [46], and it has been found that increasing visual feedback may aggravate tremor. It also has been found that expect for CTC, striatum (V3/V5) and IPL are related to the severity of tremor and pointed out that ET is not only manifested as tremor, but also as a variety of non motor manifestations, including cognitive impairment, depression, anosmia and possible increased risk of falls, which is consistent with the findings of this paper [47]. More than that, the paper also found the abnormalities related to cognition, language, hearing, memory, vision, smell and spatial cognition.

Although some results have been identified in our study, it can not be denied that there are some limitations in the paper. Firstly, in the stage of model-level and feature-level assessment, we have achieved superior classification performance. However, since maybe there are limited number of samples, 100% accuracies have been obtained. Therefore, it is necessary to collect sufficient samples of PD and ET patients to verify the founds in the paper further. Secondly, we have only investigated the classification results on GM, multi-modality and multi-center data will be helpful find more meaningful and interesting results from different aspects in the future.

## Conclusion

In the paper, we have proposed a machine learning framework to identify the biomarkers of ET and PD and classify ET from PD. To the best of our knowledge, this is the first study to investigate the differences between ET and PD via machine learning techniques based on structural MRI. Although the viability and effectiveness of the framework in clinical surroundings still need to be confirmed, a few important findings have been obtained. First of all, this study has confirmed the widespread alterations of GM for ET patients, and the large overlap of biomarkers between ET and PD from the perspective of neuroimaging. Furthermore, an excellent classification performance has been achieved to discern ET and PD. These findings in the paper have demonstrated that the different abnormal patterns of ET and PD on structural MRI could provide helpful information to classify ET and PD based on machine learning. Future studies are warranted to investigate the potential application of machine learning based on larger dataset in clinical diagnosis of ET and PD.

## Methods

### Statistical analysis

In this study, we have used two-sample *t*-test on PD and NC, ET and NC, respectively. Meanwhile, we also have regressed age, gender and total intracranial volume (TIV) during a two-sample *t*-test to remove the effects of covariates. Furthermore, we have adopted statistical significance $p<0.001$ (uncorrected) and the cluster size larger than and equal to 50 to detect group differences and compare with the results of machine learning.

### The proposed MVPA framework

**Fig. 9:**
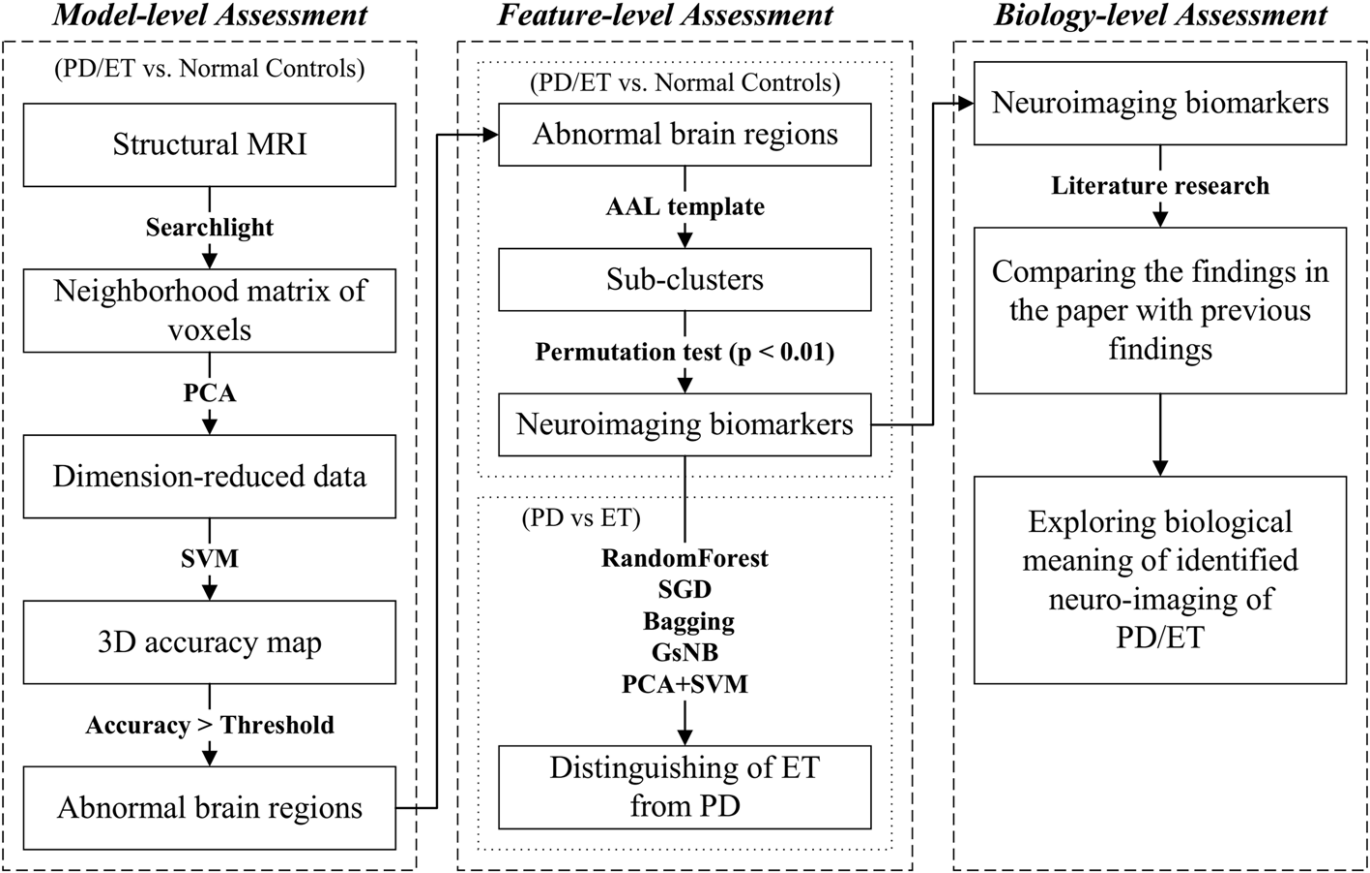
The proposed machine learning framework

During neuroimaging studies, the searchlight technique has been regarded as the most intuitively appealing approaches and often integrated with dimensionality reduction technologies when implementing MVPA. The searchlight analysis have been applied to discover local and intrinsic information presented in structural/functional MRI [48–50]. But searchlight methods also have some limitations to cause serious classification errors in practice, for example, it probably misidentifies a cluster as informative and fails to detect informative voxels. It has been suggested to conduct confirmatory and sensitivity tests to avoid the distorted results [51]. Consequently, we have conducted searchlight-based machine learning technique and processed permutation test based on prior structural brain atlas (Automated Anatomical Labeling: AAL) to relieve edge effects which lead to that the boundaries of brain regions can be recognized as informative clusters easily. The whole framework is illustrated in Fig. 9.

During model-level assessment, for two-class classification problems (PD vs. NC /ET vs. NC), we have used the searchlight technique to obtain a data matrix from a sliding spherical window centered with a specific voxel with the radius of 2 mm, which has been input principal component analysis (PCA) to extract meaningful features. At last, Support Vector Machine (SVM) have been applied to classify patients (PD/ET) from NC. In our study, we have used PCA to reduce the dimensions of data according to retaining 80% of the variance rather then to assigning the fixed number of features [52]. The average classification accuracy based on fivefold cross-validation has been considered as the discriminant capability of the centered voxel. Finally, several clusters have been obtained based on a 3D accuracy map with a specific threshold (larger than 70%) in the paper, which can be considered as distinguished brain regions with excellent classification performance roughly.

During feature-level assessment, considering edge effects of brain regions in structural MRI, the paper has separated these clusters into different sub-clusters based on AAL template and conducted permutation test on each sub-clusters to evaluate its robustness of classification performance. For permutation test, let $D=\left\{ \left( X_{i}, y_{i}\right) \right\} _{i=1}^{n}$, where $X_{i}$ is the original data and $y_{i}$ is the corresponding labels, and let ${\widehat{D}}$ be a set of *k* randomized version of the original data *D* sampled from a given null distribution. The empirical *p*-value for the classifier *f* is calculated as follows [53]:

1 $$\begin{aligned} p=\frac{\left| \left\{ \mathrm{{for}}\, D^{\prime } \in {\widehat{D}}: \mathrm{{acc}} \left( f, D^{\prime }\right) \ge \mathrm{{acc}}(f, D)\right\} \right| +1}{k+1}, \end{aligned}$$

where $\mathrm{{acc}}\left( f, D^{\prime }\right)$ and $\mathrm{{acc}}\left( f, D\right)$ represent the accuracies of the class *f* on data $D^{\prime }$ and *D*, respectively. The empirical *p*-value represents the fraction of randomized samples where the classifier behaved on random data better than on original data, which demonstrates how likely the accuracy obtained from the original data by chance. Therefore, if the *p*-value is small enough, we can say the null hypothesis is rejected and the discriminant capability of original data is powerful. In the paper, $p\le 0.01$ and cluster size $\ge 50$ have been used to reserve distinguished sub-clusters, which can be considered as neuro-imaging markers of PD/ET.

During biology-level assessment, we have compared the findings in the paper with previous findings to explore the biological meaning of identified neuro-imaging of PD/ET in the discussion.

### Performance evaluation

The performance of the learning model was evaluated by calculating the *sensitivity*, *specificity*, and *accuracy*, which were defined as:

2 $$\mathrm{Sensitivity}= \frac{\mathrm{TP}}{{\mathrm{TP}}+{\mathrm{FN}}},$$

3 $$\mathrm{Specificity}= \frac{\mathrm{TN}}{\mathrm{TN}+\mathrm{FP}},$$

4 $$\mathrm{Accuracy}= \frac{\mathrm{TP}+\mathrm{TN}}{\mathrm{TP}+\mathrm{FP}+\mathrm{FP}+\mathrm{FN}}.$$

Suppose *N* represents the number of patients (ET/PD), and *M* refers to the number of NC. $N^{\prime }$ is the number of patients correctly classified, and $M^{\prime }$ is the number of NC correctly classified. TP, TN, FP, and FN are represented as follows:

5 $$\begin{aligned} \mathrm{TP}= & {} \frac{N^{\prime }}{N}, \end{aligned}$$

6 $$\begin{aligned} \mathrm{TN}= & {} \frac{N-N^{\prime }}{N}, \end{aligned}$$

7 $$\begin{aligned} \mathrm{FP}= & {} \frac{M^{\prime }}{M}, \end{aligned}$$

8 $$\begin{aligned} \mathrm{FN}= & {} \frac{M-M^{\prime }}{M}. \end{aligned}$$

Accuracy, calculated as an arithmetic mean of sensitivity and specificity, was used to measure the overall performance of the model in classification. In general, sensitivity and specificity affect each other, and an increase in one of them will inevitably lead to a decrease in the other.

In addition, the area under ROC curve (AUC) is also used to evaluate the performance of classification model. The models with a AUC value closer to 1 have better classification performance:

9 $$\begin{aligned} \mathrm{AUC}=\frac{\sum _{i \in \mathrm {positiveClass}} {\mathrm {rank}}_i-\frac{M(1+M)}{2}}{M \times N}. \end{aligned}$$

## Data Availability

The datasets analyzed in this study are available from the corresponding author on reasonable request.
